# Supplementary figures and images for: DNA-damage induced cell death in yap1;wwtr1 mutant epidermal basal cells
Source: eLife. 2022 May 30;11:e72302. doi: 10.7554/eLife.72302 (PMC9197390; doi:10.7554/eLife.72302)

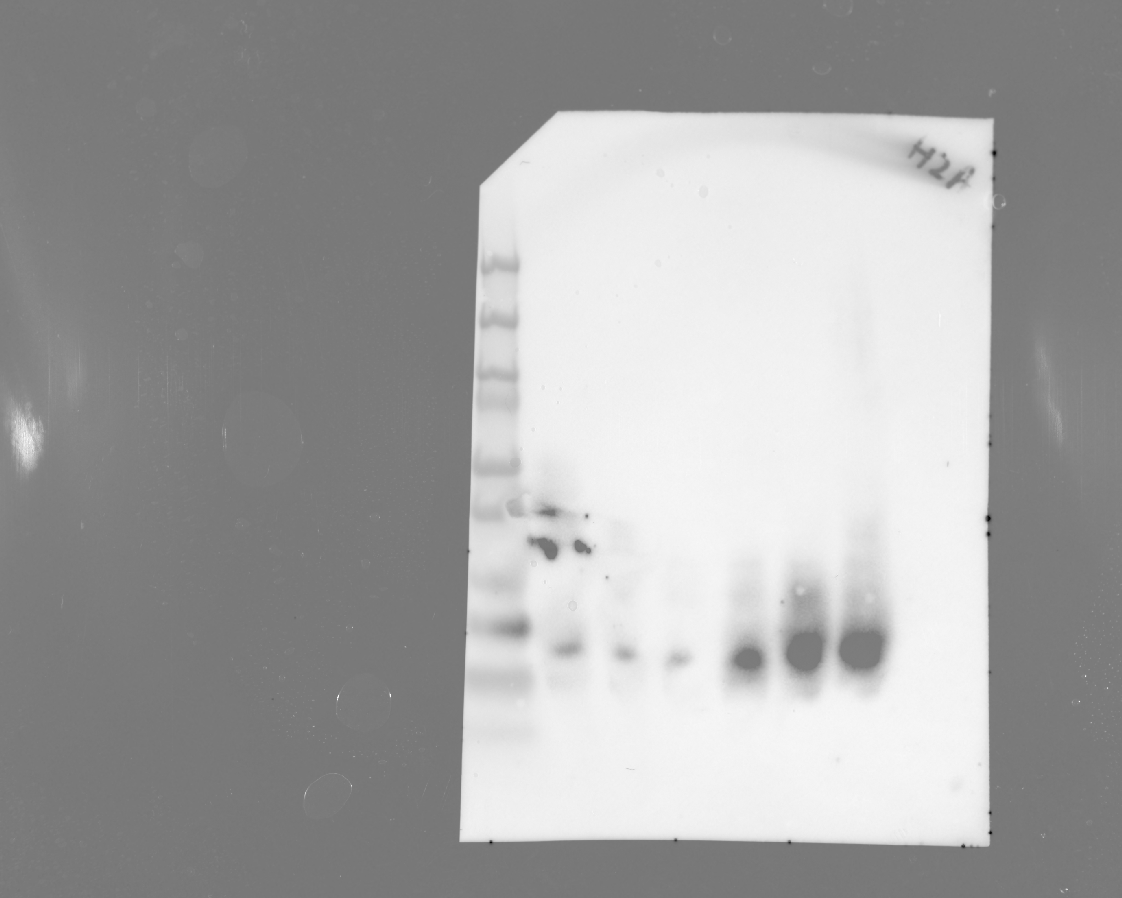

Supplement: Figure 4—figure supplement 1—source data 1. [file elife-72302-fig4-figsupp1-data1.zip › Figure4_figuresupplement1B_gH2AX.tif]

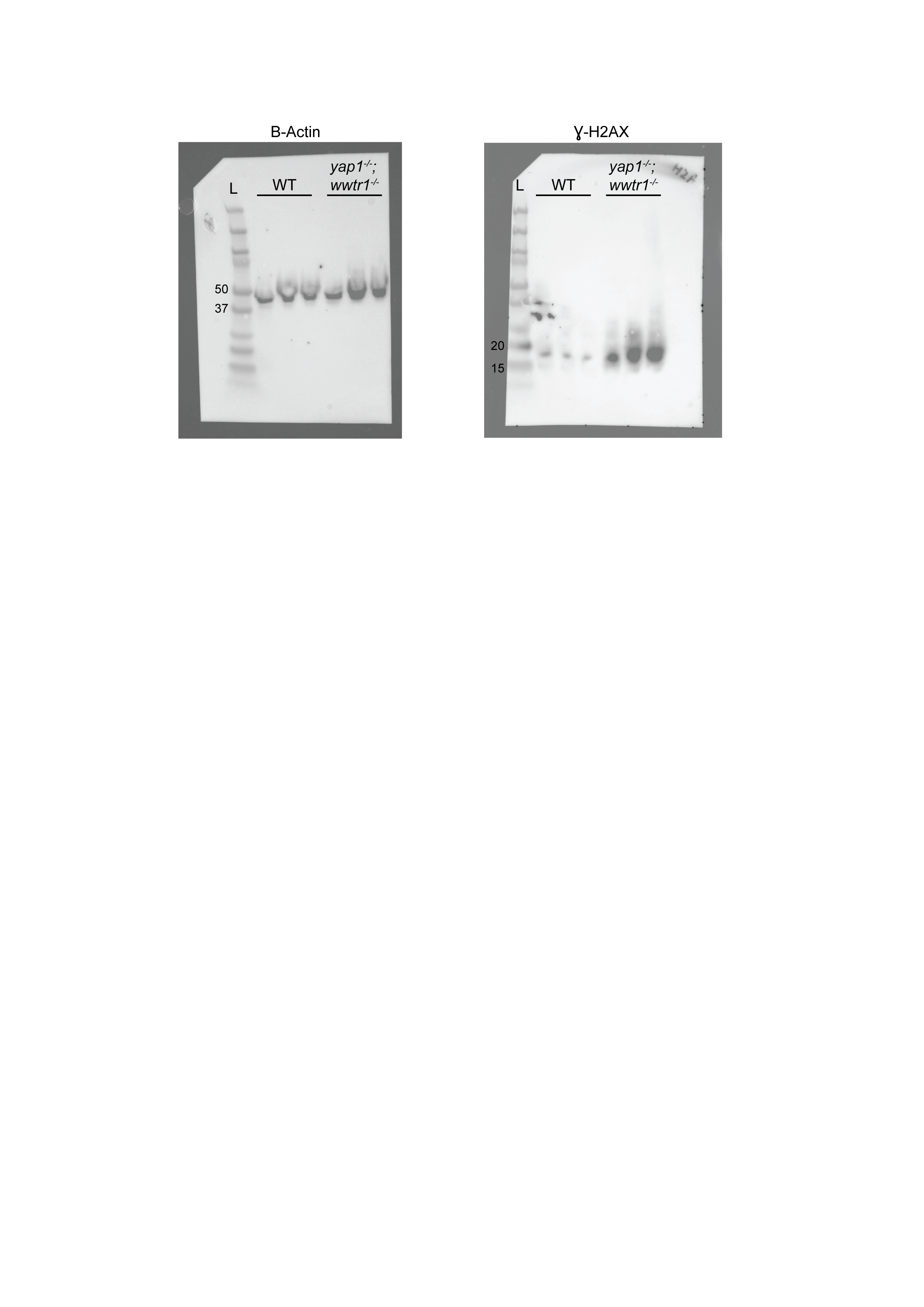

Supplement: Figure 4—figure supplement 1—source data 1. [file elife-72302-fig4-figsupp1-data1.zip › Figure_4_figuresupplement1B.tif]

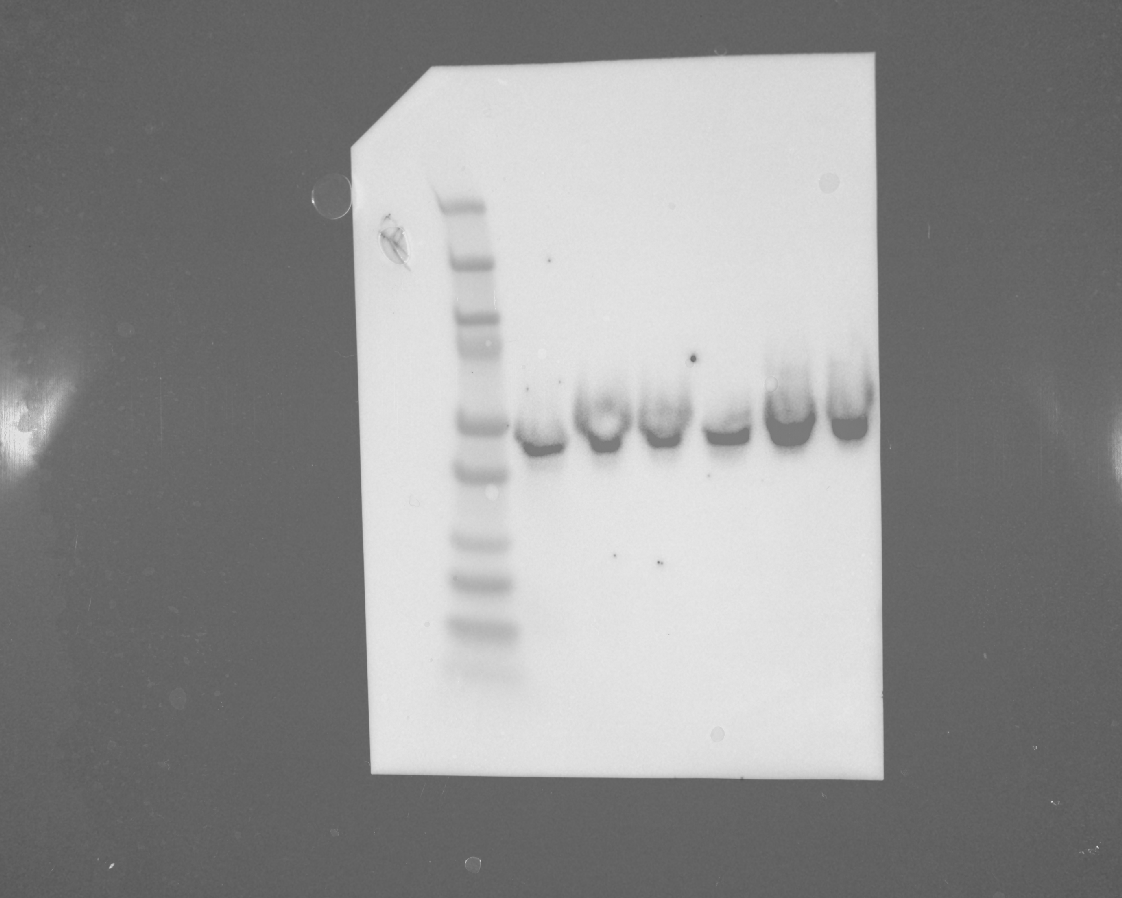

Supplement: Figure 4—figure supplement 1—source data 1. [file elife-72302-fig4-figsupp1-data1.zip › Figure4_figuresupplement1B_Bactin.tif]
